# Integrating plant physiology into simulation of fire behavior and effects

**DOI:** 10.1111/nph.18770

**Published:** 2023-02-14

**Authors:** L. Turin Dickman, Alexandra K. Jonko, Rodman R. Linn, Ilkay Altintas, Adam L. Atchley, Andreas Bär, Adam D. Collins, Jean‐Luc Dupuy, Michael R. Gallagher, J. Kevin Hiers, Chad M. Hoffman, Sharon M. Hood, Matthew D. Hurteau, W. Matt Jolly, Alexander Josephson, E. Louise Loudermilk, Wu Ma, Sean T. Michaletz, Rachael H. Nolan, Joseph J. O'Brien, Russell A. Parsons, Raquel Partelli‐Feltrin, François Pimont, Víctor Resco de Dios, Joseph Restaino, Zachary J. Robbins, Karla A. Sartor, Emily Schultz‐Fellenz, Shawn P. Serbin, Sanna Sevanto, Jacquelyn K. Shuman, Carolyn H. Sieg, Nicholas S. Skowronski, David R. Weise, Molly Wright, Chonggang Xu, Marta Yebra, Nicolas Younes

**Affiliations:** ^1^ Earth & Environmental Sciences Division Los Alamos National Laboratory Los Alamos NM 87545 USA; ^2^ San Diego Supercomputer Center and Halicioglu Data Science Institute University of California San Diego La Jolla CA 92093 USA; ^3^ Department of Botany University of Innsbruck 6020 Innsbruck Austria; ^4^ Ecologie des Forêts Méditerranéennes (URFM) INRAe 84914 Avignon France; ^5^ USDA Forest Service Northern Research Station New Lisbon NJ 08064 USA; ^6^ Tall Timbers Research Station Tallahassee FL 32312 USA; ^7^ Department of Forest and Rangeland Stewardship Colorado State University Fort Collins CO 80523 USA; ^8^ Rocky Mountain Research Station USDA Forest Service Missoula MT 59801 USA; ^9^ Department of Biology University of New Mexico Albuquerque NM 87131 USA; ^10^ Southern Research Station USDA Forest Service Athens GA 30602 USA; ^11^ Department of Botany and Biodiversity Research Centre The University of British Columbia Vancouver BC V6T 1Z4 Canada; ^12^ Hawkesbury Institute for the Environment Western Sydney University Penrith NSW 2753 Australia; ^13^ NSW Bushfire Risk Management Research Hub Wollongong NSW 2522 Australia; ^14^ School of Life Sciences and Engineering Southwest University of Science and Technology Mianyang 621010 China; ^15^ Department of Crop and Forest Sciences and JRU CTFC‐AGROTECNIO Universitat de Lleida Lleida 25198 Spain; ^16^ Fire and Resource Assessment Program California Department of Forestry and Fire Protection South Lake Tahoe CA 96155 USA; ^17^ Environmental Protection and Compliance Division Los Alamos National Laboratory Los Alamos NM 87545 USA; ^18^ Environmental and Climate Sciences Department Brookhaven National Laboratory Upton NY 11973 USA; ^19^ Climate and Global Dynamics Laboratory, Terrestrial Sciences Section National Center for Atmospheric Research Boulder CO 80305 USA; ^20^ Rocky Mountain Research Station USDA Forest Service Flagstaff AZ 86001 USA; ^21^ Northern Research Station USDA Forest Service Morgantown WV 26505 USA; ^22^ Pacific Southwest Research Station USDA Forest Service Riverside CA 92507 USA; ^23^ Cibola National Forest USDA Forest Service Albuquerque NM 87113 USA; ^24^ Fenner School of Environment and Society Australian National University Canberra ACT 2601 Australia; ^25^ School of Engineering Australian National University Canberra ACT 2601 Australia

**Keywords:** carbon dynamics, fire behavior, fire effects, fire modeling, plant physiology, remote sensing, vegetation–fire interactions, water dynamics

## Abstract

Wildfires are a global crisis, but current fire models fail to capture vegetation response to changing climate. With drought and elevated temperature increasing the importance of vegetation dynamics to fire behavior, and the advent of next generation models capable of capturing increasingly complex physical processes, we provide a renewed focus on representation of woody vegetation in fire models. Currently, the most advanced representations of fire behavior and biophysical fire effects are found in distinct classes of fine‐scale models and do not capture variation in live fuel (i.e. living plant) properties. We demonstrate that plant water and carbon dynamics, which influence combustion and heat transfer into the plant and often dictate plant survival, provide the mechanistic linkage between fire behavior and effects. Our conceptual framework linking remotely sensed estimates of plant water and carbon to fine‐scale models of fire behavior and effects could be a critical first step toward improving the fidelity of the coarse scale models that are now relied upon for global fire forecasting. This process‐based approach will be essential to capturing the influence of physiological responses to drought and warming on live fuel conditions, strengthening the science needed to guide fire managers in an uncertain future.

**Table:** 

	Contents	
	Summary	953
I.	Introduction	953
II.	Existing models of fire behavior and effects	955
III.	Physiological controls of fire behavior and effects	956
IV.	Integrating fine‐scale physiology with fire models	961
V.	Remote sensing of plant water and carbon to inform process modeling	963
VI.	Conclusions and future directions	964
	Acknowledgements	965
	References	966

## I. Introduction

Wildland fire burns hundreds of millions of hectares of forests, woodlands, and grasslands annually (Giglio *et al*., 2013), shaping terrestrial ecosystems (Bond & van Wilgen, 1996; Bond & Keeley, 2005; Bond, 2021), and their impacts on global carbon (Bowman *et al*., 2009) and water (Li & Lawrence, 2017) cycles. To accurately predict fire behavior and resulting effects, we need to understand the influences of vegetation structure and physiology on combustion (e.g. Byram, 1959; Weise & Wright, 2013) and the microenvironment (e.g. Finnigan, 2000; Banerjee *et al*., 2020; Atchley *et al*., 2021). Recent studies demonstrate that burned area shows greater increases in forests and shrublands where vegetation moisture is more sensitive to water limitation (Rao *et al*., 2022), and that hydraulic traits of woody evergreen species are responsible for up to 3.6‐fold variation in live fuel moisture content (LFMC, ratio of biomass water content to oven‐dry biomass, Table 1; Scarff *et al*., 2021). These findings emphasize the importance of accounting for ecophysiological controls on woody vegetation in wildfire forecasting and prescribed fire planning, where fuel effects dominate under less extreme fire danger conditions (Cruz *et al*., 2022). This will be particularly important in fire‐susceptible ecosystems which experience significant drought and LFMC declines below thresholds that drive increases in fire behavior (Pimont *et al*., 2019a). As atmospheric aridity continues to increase with rising temperatures, compounding plant water stress (Grossiord *et al*., 2020), the number of regions for which live fuels (i.e. living plants) are important in determining the behavior of fires and their ecological outcomes is likely to expand (Resco de Dios *et al*., 2021).

**Table 1:** Glossary of pyro‐ecophysiology terms.

Canopy bulk density (CBD, mass per unit volume)	Measure of how closely canopy fuels are packed, reflects likelihood that fire can move through the forest canopy
Carbon starvation	Plant mortality resulting from inability of *NSC* to meet metabolic demands
Cavitation	Process by which excessive water tension causes expansion of dissolved air to form bubbles; in plants, this causes a break in the water column and a decrease in *hydraulic conductivity*
Conduction	Heat transfer through a material from a region of higher temperature to a region of lower temperature
Consumption (mass per unit area)	Amount of biomass consumed during fire
Convection	Aeat transfer by the movement of a gas or liquid
Embolism	Blockage of a vessel by a mass; in plants, caused by air bubbles formed in xylem *via cavitation*
Equivalent water thickness (EWT, g m^−2^)	Measure of leaf water content
Fire radiative energy density (FRED, MJ m^−2^)	Measure of the intensity of radiative energy released from fuel during a fire
Hydraulic conductivity (water mass or volume per unit time per unit area)	Measure of a system's ability to transport water
Hydraulic failure	Plant mortality resulting from failure of the water column by exceeding *PLC* thresholds
Leaf mass per area (LMA, g m^−2^)	Measure of leaf thickness and density; inverse of specific leaf area; also called dry matter content (DMC) in remote sensing applications
Live fuel moisture content (LFMC, %)	Ratio of water mass to dry mass in living plants; controlled largely by physiological mechanisms, rather than weather
Non‐structural carbohydrates (NSC)	Plant carbon used for functions other than building structural biomass, such as growth, metabolism, osmoregulation, transport, storge, and defense
Osmotic potential (MPa)	Potential of water molecules to move from a less concentrated to a more concentrated solution across a semi‐permeable membrane
Percent loss of conductivity (PLC)	A measure of xylem vulnerability to cavitation at a given *water potential*
Phenology	Biological cycles resulting from seasonal or interannual climate variations
Physiology	Bynamic chemical and physical processes that govern function
Pyro‐ecophysiology	Ecophysiology‐based approach to live fuel research that considers how plant water and carbon cycles independently and collectively interact at the leaf and whole plant level to regulate flammability and subsequent fire behavior (Jolly & Johnson, 2018)
Radiation	Heat transfer through a gas or vacuum other than by heating of the intervening space
Relative water content (RWC, %)	Plant water content relative to its fully hydrated state
Senescence	Process of biological aging; can be stress‐induced or developmental
Traits	Morphological, physiological or phenological features measurable at the individual level (Violle *et al*., 2007)
Transpiration	Loss of water vapor from a living body; in plants, evaporation *via* stomata
Vapor pressure deficit (VPD, kPa)	The difference between the amount of moisture in the air and how much moisture the air can hold when saturated; measure of atmospheric aridity
Water potential (MPa)	Pressure potential required to remove a water molecule from its matrix (e.g. xylem); a measure of plant water status

Historically, representation of live fuels in fire behavior models has been limited to static fuel models (Table 2) that generalize vegetation into classes, such as grass, shrub, and timber (Albini, 1976; Scott & Burgan, 2005; but see Rothermel & Philpot, 1973; Hough & Albini, 1978 for seasonally‐ and age‐dynamic fuel models). With recent advances in process‐based modeling and remote sensing of both fuels and fires, there is now opportunity to capture more realistic fuel heterogeneities, including the physiological dynamics that determine live fuel conditions (‘pyro‐ecophysiology’, Jolly & Johnson, 2018). This will allow exploration of their influence on fire behavior, ecological outcomes (i.e. plant injury, mortality, and recovery; hereafter, ‘fire effects’) and cascading hazards. Recent work has called for modeling efforts to improve integration of fire behavior and its effects (e.g. Hood *et al*., 2018; O'Brien *et al*., 2018; Kleynhans *et al*., 2021) to better capture fire–vegetation–environment feedbacks. This integration is particularly important in the context of low‐intensity and prescribed fire. While fuel heterogeneity has little effect in a high‐intensity fire environment (Atchley *et al*., 2021; Cruz *et al*., 2022), changes in phenology can make the difference between containment and escape for prescribed fires, where combustion dynamics are engineered to achieve desired biological and physical outcomes (e.g. species‐specific vegetation survival or mortality, fire risk reduction, soil protection, and smoke management (Hiers *et al*., 2020)) with implications for future fire and landscape dynamics (Mitchell *et al*., 2009; Gallagher *et al*., 2021). Plant water and carbon cycles have been recognized as important to both fire behavior (Nelson, 2001; Macias Fauria *et al*., 2011; Jolly *et al*., 2014; Jolly & Johnson, 2018) and ecological fire effects (Michaletz & Johnson, 2007; Hood *et al*., 2018; Bär *et al*., 2019). Yet, they have not been considered as an integrating framework mechanistically linking the two.

**Table 2:** Glossary of models and remote sensing methods.

Models	
3D Fuel frameworks	Models that generate synthetic 3D fuels as inputs for *CFD* fire models
Computational fluid dynamics (CFD)	Physics‐based models that simulate the interaction of liquids and gases based on fluid mechanics principles; used in simulation of fine‐scale fire behavior and fire–atmosphere interactions within individual stands on timescales of minutes to hours
Ecosystem process	Mechanistic models based on theoretical understanding of ecological processes; for fire applications, can represent both fire behavior and effects on regional to global scales
Empirical fire effects	Statistical models that predict tree status (live, dead) as a function of tree characteristics and observed fire injury, often estimated based on flame length
Fire danger rating systems	Broad‐scale assessments of fire ignition, spread, and hazard potential based on current and antecedent weather, fuels, and topography
Functional structural plant models (FSPMs)	Mechanistic models of 3D plant structure, environment, and physiological response, from gene to community scales
Fuel models	Stylized set of fuel bed characteristics used in fire models
Landscape fire succession	Spatial simulation models of fire and vegetation dynamics at stand to landscape scales
Operational fire	Computationally inexpensive models that rely on empirical representations of fire behavior and fixed, stylized *fuel models* to determine flame length and resulting mortality
Process‐based fire effects	Models which predict injury to different plant compartments based on heat transfer
Vegetation demography	Models which capture dynamic, size‐structured vegetation
Remote sensing	
Hyperspectral	Acquired in narrow, contiguous wavelength bands; high spectral resolution allows for material characterization, inference of chemical/biological processes, and novel signature identification
Lidar	Light detection and ranging; active *optical* sensing method using return time of a pulsed laser to measure distance
Microwave	Detects backscattering of actively transmitted radiation in the microwave (1 cm^–1^ m) portion of the electromagnetic spectrum; longer wavelengths allow penetration of clouds, rain, and surfaces
Multispectral	Acquired in broad, often discontinuous, wavelength bands; low spectral resolution reduces cost and complexity for monitoring known signatures
Optical	Detects *reflected* radiation in the visible (VIS, 380–780 nm), near‐ (NIR, 780–1000 nm) and short‐wave infrared (SWIR, 1000–2500 nm) portions of the electromagnetic spectrum; primarily passive
Thermal	Detects *emitted* radiation in the thermal infrared (TIR, 780 nm^–1^ mm) portions of the electromagnetic spectrum; primarily passive

**Fig. 1:**
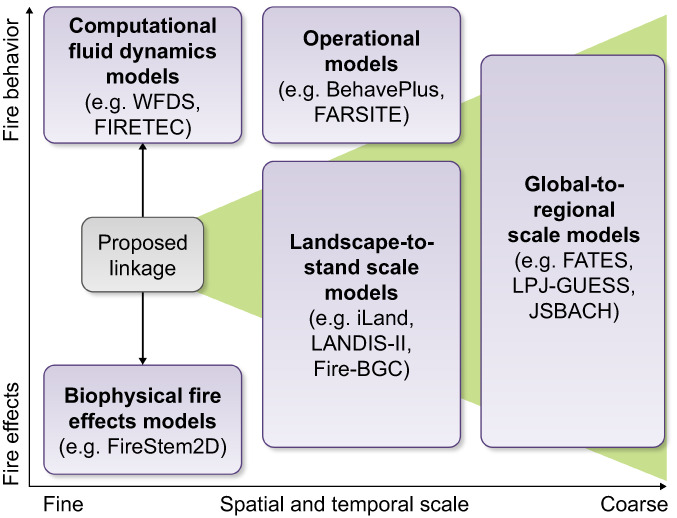
Model classification according to scale and process representation. In this conceptual figure, we show the classification applied to different models of fire behavior and effects discussed in this review. We also highlight our proposed linkage between fine‐scale computational fluid dynamics (CFD) and biophysical fire effects models.

We argue that plant water and carbon cycles drive live fuel moisture and dry mass dynamics, which influence heat transfer into the plant during a fire and subsequent postfire survival and recovery. This focus on plant water and carbon dynamics provides a mechanistic framework that links plant physiology to fine‐scale fire behavior and fire‐induced effects on plant tissues, addressing a gap in current approaches to modeling fire and vegetation (Fig. 1). We focus in this review on the interactions of woody plants and fire, due to the availability of literature and to maintain focus on the underlying argument. However, many of the needs and underlying connections that we recognize have importance to nonwoody systems. Understanding how within‐plant physiology and fire interact will allow exploration of mechanisms important to ensuing plant, stand, and landscape‐level vegetation dynamics, which influence subsequent fire behavior. To fully capture fire‐ecosystem feedbacks, we propose exploring new ways to bring together fire behavior and effects models at fine scales where both fire and physiological processes can be represented in sufficient detail to define important biophysical mechanisms, with heat transfer to vegetation providing the mechanistic linkage from physiology to fire behavior and subsequent effects (Varner *et al*., 2021). A fundamental understanding of how fire and plants interact at the fine scale is needed to constrain simulations used at management‐relevant scales. Under unprecedented future climate conditions, for which we do not have past or present analogs, biophysical process modeling will be essential to understand response to conditions that fall outside the range of variability captured by current empirical models. Linking remotely sensed estimates of plant water and carbon status to models of fire behavior and effects offers a mechanistic approach critical to capturing the influence of physiological responses to drought and warming on live fuel conditions under global change.

## II. Existing models of fire behavior and effects

There are many existing fire behavior and effects models under use and continuous development. Rather than focusing on any one model or group of models, here we focus on commonly applied scales and model characteristics. We classify these as global to regional‐scale models, landscape to stand‐scale models, operational models, computational fluid dynamics models, and fine‐scale biophysical fire effects models.

On global to regional scales (Fig. 1), fire behavior and effects have been combined within ecosystem process models using various approaches to simulate ecosystem dynamics with fire disturbance (see Rabin *et al*., 2017 for in‐depth review). Simulations commonly span decades to centuries and may include the entire globe. In these models, fire behavior and effects are commonly simulated explicitly at the computational grid level (*c*. 0.1–1°) or implicitly at a subgrid level. Fire spread is a function of fuel characteristics (loading, size distribution, and moisture), ignition patterns, weather, and latent suppression. These models often capture a dimension of vegetation growth and/or demographics which can dynamically inform fuel loads and fuel availability. Fire behavior is usually represented empirically based on experimental and modeling studies by Rothermel (1972) and Van Wagner (1973). Fire effects are determined by total energy release and thresholds for mortality by either individuals, cohorts, or by fractioning existing vegetation. For example, recent advances in vegetation demography modeling include the addition of plant hydraulics in FATES (Christoffersen *et al*., 2016), allowing decoupling of water and carbon dynamics for more direct determination of live fuel vulnerability to fire at multiple timescales. Live fuel moisture content dynamics are captured by simulating water‐ and carbon‐cycle processes directly at subdaily time scales, allowing for assessment of climate impacts (e.g. change in CO_2_, temperature, and precipitation) on future LFMC trends under different climate scenarios (Ma *et al*., 2021). However, simplified representation of fire precludes mechanistic understanding of fire–physiology interactions.

At stand to landscape scales, a large number of models were developed for spatial plant ecology and fire occurrence at the scale relevant to forest management (10–100 km^2^; e.g. FIRE‐BGC (Keane *et al*., 2004), LANDIS‐II (Sturtevant *et al*., 2009; Scheller *et al*., 2019)). These models are applied to decadal to century‐long simulations. Observed or modeled fire dynamics account for stand‐to‐stand fire spread probabilistically or through the estimation of mechanistic processes. These models simulate cohorts or representative sets of individual plants/trees to estimate vegetation development and demography which inform fuel loading and availability. While this class of models can be leveraged to explore fire behavior and effects, they do not mechanistically link the two (Keane *et al*., 2004). Within the stand (1–10 km^2^), fire effects are usually homogenous or stochastically based on homogenous traits of the stand (Furniss *et al*., 2022). For example, FIRE‐BGCv2 (Keane *et al*., 2011) links biogeochemical processes with stand‐scale fire behavior and effects based on live and dead fuel loading, leveraging FARSITE (Finney, 1998), but does not address within‐stand variability. Keane *et al*. (2004) and Sturtevant & Fortin (2021) provide extensive reviews of landscape to stand‐scale models.

Operational fire models such as BehavePlus (Andrews, 2013), FARSITE, FLAMMAP (Finney, 1998), or FFE‐FVS (Reinhardt & Crookston, 2003) also represent fire behavior and effects. However, these models, which need to be computationally inexpensive to be useful, rely on empirical representations of fire behavior (Rothermel, 1972), and use fixed, stylized fuel models (Scott & Burgan, 2005) to determine flame length and resulting mortality. Their linkage between fire behavior and effects is thus very simple, and largely does not account for dynamic plant physiology or demography.

At the substand scale (< 1 m–1 km), the most advanced representations of fire behavior and effects are currently found in distinct classes of models. Fire behavior is best captured with Computational Fluid Dynamics (CFD) models such as FIRETEC (Linn *et al*., 2002) or the Wildland–Urban Interface Fire Dynamics Simulator (WFDS, Mell *et al*., 2009). These models simulate individual fire events, or portions thereof, on short timescales of minutes to hours. Fuel parameters are static, with exception of moisture mass and dry mass of the fuel, which may decrease through dehydration and consumption, respectively. These models do not represent fire effects, as they do not explicitly distinguish between live and dead fuels, or between different vegetation species or functional groups. In absence of explicit vegetation response to changing meteorological conditions, they cannot resolve changes in LFMC or the sensitivity of fire behavior to live fuel moisture dynamics (Jolly, 2007).

Prediction of fine‐scale ecological fire effects (postfire vegetation injury, mortality, and recovery) has been approached using both empirical and process‐based models, with fire intensity provided as input from other models or measurements. Most postfire mortality models rely on empirical correlations (Woolley *et al*., 2012; Hood *et al*., 2018) which predict vegetation status (live or dead) as a function of plant characteristics (e.g. species, bark thickness, height, and diameter) and observed fire injury (e.g. crown scorch, and bark char), often estimated based on flame scorch height (Van Wagner, 1973). A number of process‐based biophysical fire effects models predict injury to different woody plant compartments (e.g. stem (FireStem2D, Chatziefstratiou *et al*., 2013) or crown (Michaletz & Johnson, 2006) heating models). Few, however, have considered injuries to more than one part of the plant (Michaletz & Johnson, 2008), and none address the influence of interacting injuries across multiple woody plant compartments on postfire plant function.

Direct coupling of fine‐scale fire behavior and effects models remains challenging due to the different temporal scales that are relevant for both. As an alternative to solve this challenge, we propose exploring nested modeling frameworks (Gettelman *et al*., 2022; Shuman *et al*., 2022) which can transfer information across models at scales where both fire and physiological processes can be represented in sufficient detail to define important biophysical mechanisms. Recent work has made strides in mechanistically linking fire behavior to ecological effects through plant traits (Zylstra, 2021), but without mechanistic determination of vegetation density and moisture content resulting from plant water and carbon dynamics. By incorporating fine‐scale physiology, we can capture dynamic water and carbon to represent these properties more realistically.

## III. Physiological controls of fire behavior and effects

### 1. Evidence for the role of plant water and carbon in fire behavior

**Fig. 2:**
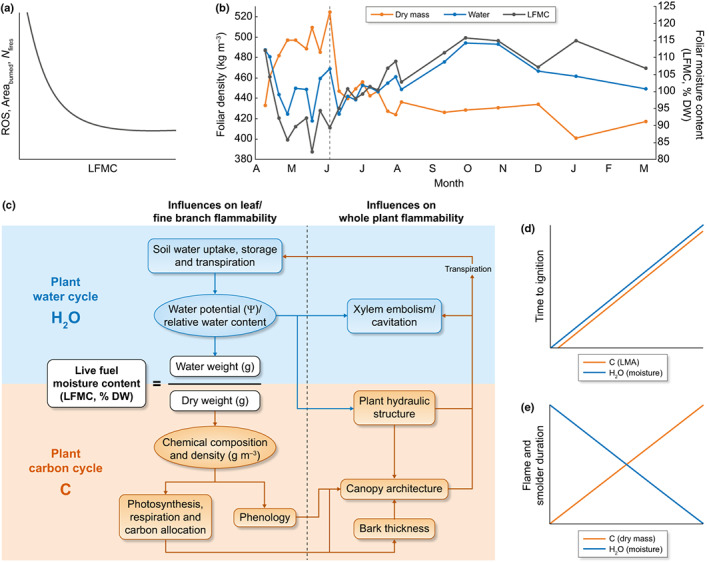
Live fuel moisture content (LFMC, black), an important driver of landscape‐scale fire behavior (a), is a function of plant carbon (C, orange) and water (H_2_O, blue) (b, c), which can have opposing influences on fire behavior in leaf level burn experiments (d, e). (a) Rate of spread (ROS), area burned, and fire occurrence (*N*_fires_) as a function of LFMC, as shown by Dennison & Moritz (2009), Martin‐StPaul *et al*. (2018), Pimont *et al*. (2019a,b), and others. (b) Monthly variation in foliar dry mass (orange) and water (blue) density, and foliar moisture content (dry mass/water mass, LFMC; black) extracted from Jolly *et al*. (2016) with WebPlotDigitizer (Rohatgi, 2021). Dashed vertical line indicates divergence between LFMC and water density driven by increasing dry mass. (c) Conceptual model of LFMC as a function of plant water and carbon cycles redrawn from Jolly & Johnson (2018). (d) Time to ignition as a function of leaf carbon (leaf mass per area, LMA) and water (moisture) as shown in Grootemaat *et al*. (2015) and Bianchi *et al*. (2019). (e) Flame and smolder duration as a function of leaf carbon (dry mass) and water (moisture), as shown in Grootemaat *et al*. (2015) and Bianchi *et al*. (2019).

Fire behavior is broadly controlled by complex interactions among fuel density and moisture content, topography, and the atmosphere (Countryman, 1966). These factors not only interact, but also can be highly variable in space and time, resulting in dynamic fire environments. Live fuel moisture content is a key fuel property governing fire behavior (Fig. 2a). Declining LFMC is associated with an increase in area burned (Dennison & Moritz, 2009; Nolan *et al*., 2016; Pimont *et al*., 2019b), and LFMC below 100% sharply increases fire rate of spread (ROS, Pimont *et al*., 2019a) and the probability of large fires (Martin‐StPaul *et al*., 2018) (Fig. 2a). Given its impact on fire behavior, LFMC has been incorporated into fire danger rating systems (e.g. Deeming *et al*., 1977; Stocks *et al*., 1989) and fire behavior models (e.g. Rothermel, 1972).

The water and dry mass components of LFMC are outcomes of plant water‐ and carbon‐cycle processes, respectively, and are controlled by environmental conditions, soil properties, and plant physiology (Fig. 2b,c; Macias Fauria *et al*., 2011; Jolly & Johnson, 2018; Ruffault *et al*., 2018). Plant water and carbon can vary independently in both space and time, with opposing effects on LFMC. For example, a sharp prebudburst (early June) increase in foliar dry mass of older, live pine needles has been shown to reduce LFMC despite increasing foliar water mass (Fig. 2b; Jolly *et al*., 2014, 2016). Based on these findings, Jolly & Johnson (2018) proposed a framework decomposing LFMC into plant water and carbon cycle processes (Fig. 2c), where the numerator is determined by whole plant hydraulics (e.g. transpiration; relative water content; and xylem embolism), and the denominator by carbon allocation (e.g. chemical composition/density; phenology; and canopy architecture). These processes influence fire behavior both by changing tissue‐ and canopy‐level properties that influence heat transfer, and by altering the proportion of dead biomass in fuels and on the ground surface. Seasonal or drought‐induced senescence and shedding are examples of physiological processes that alter leaf, fine branch, and whole plant flammability, transforming live fuels into dead fuels and increasing litter accumulation. Tissue senescence is associated with shifts in osmolytes and water content (Milla *et al*., 2007), which can increase or decrease LFMC. Tissue mortality also increases the dead to live fuel ratio, causing a strong increase in fire spread and intensity (e.g. Cruz *et al*., 2015; Sieg *et al*., 2017; Balaguer‐Romano *et al*., 2020). When senesced tissues are shed, litter accumulates in the surface fuel‐bed and may increase the likelihood of surface fire.

The importance of both plant water‐ and carbon‐cycle processes for fire behavior becomes clear when examining their effects on heat transfer and combustion (Michaletz & Johnson, 2007; Bär *et al*., 2019; Dietenberger *et al*., 2020; Kleynhans *et al*., 2021). Preheating, the process by which heat transfer evaporates water, dries, and decomposes carbon‐containing polymers in unburned fuels, occurs *via* conduction, convection, and radiation. Thermal conductivity varies with material density, temperature, and water content, while convection depends on geometry and orientation of the fuel surface. The proportion of radiation absorbed by unburned fuel depends on both its thermal absorptance as well as geometry and orientation. Fuel chemistry and water content are therefore important to both conductive and radiative heat transfer through their influence on thermal conductivity and absorptance, while convection and radiative heat transfer to the fuel surface depend on vegetation geometry and orientation, including leaf, branch, crown, or canopy structure (Michaletz & Johnson, 2006; Pausas & Moreira, 2012). The rate at which the temperature increases in response to heat transfer depends on the mass and specific heat of the biomass, which depend on plant tissue water content and composition (Boardman *et al*., 2021). Water content also determines how much energy is required to heat the fuel to evaporation and combustion temperatures (Yashwanth *et al*., 2016), while the content of carbon‐based polymers determines the amount and rate of thermal decomposition, influencing the rate of preheating and time to combustion (Kilzer & Broido, 1965). Fuel chemistry and water content are also critical in determining whether combustion reactions are self‐sustaining and how they will contribute to fire intensity and rate of spread (Quintiere, 2006; Matt *et al*., 2020). During combustion, the content of organic polymers determines how much energy can be released, and water content can determine whether flaming or smoldering combustion occurs. Higher rates of fire spread and probability of flaming combustion, which occurs at high temperature and/or low moisture, are therefore more likely when live fuels are water stressed and the ratio of water mass to dry mass (LFMC) is lower.

Results from leaf‐level burn experiments support the influence of plant water and carbon processes on preheating. Time to ignition (Fig. 2d) has been shown to increase with moisture (Grootemaat *et al*., 2015; Bianchi *et al*., 2019; but see Fletcher *et al*., 2007) and leaf mass per area (LMA, inverse of specific leaf area, Grootemaat *et al*., 2015), which is associated with accumulation of total structural carbohydrates and lignin (Poorter *et al*., 2009). Flame and smolder duration (Fig. 2e) also increase with dry mass (Grootemaat *et al*., 2015), but decrease with moisture (Bianchi *et al*., 2019). These contrasting effects of leaf‐level water and carbon on different fire behavior characteristics underscore the importance of capturing LFMC components independently. Despite evidence from Alam *et al*. (2020) that certain leaf and shoot flammability metrics (e.g. ignitibility and combustibility) are decoupled, the same study showed a positive relationship of leaf dry matter content with shoot and species‐level flammability, suggesting that effects of leaf water and carbon on fire behavior are scalable. At canopy scales, Jolly *et al*. (2016) found that increased leaf density, which accounts for changes in dry mass associated with phenological change in foliar chemistry and carbon allocation, increased modeled crown fire propagation and area burned. Additionally, canopy bulk density, a measure of canopy architecture influenced by water‐ and carbon‐cycle processes *via* growth rate, environment, and phenology, has long been recognized as an important driver of spread rate in active crown fires (Van Wagner, 1977; Resco de Dios, 2020). The independent relationships of water‐ and carbon‐cycle processes with fire behavior, and their complex interdependence, highlight the need for a more mechanistic representation of LFMC that accounts for underlying physiology and allows for dynamic change.

### 2. Evidence for the role of plant water and carbon in fire effects

**Fig. 3:**
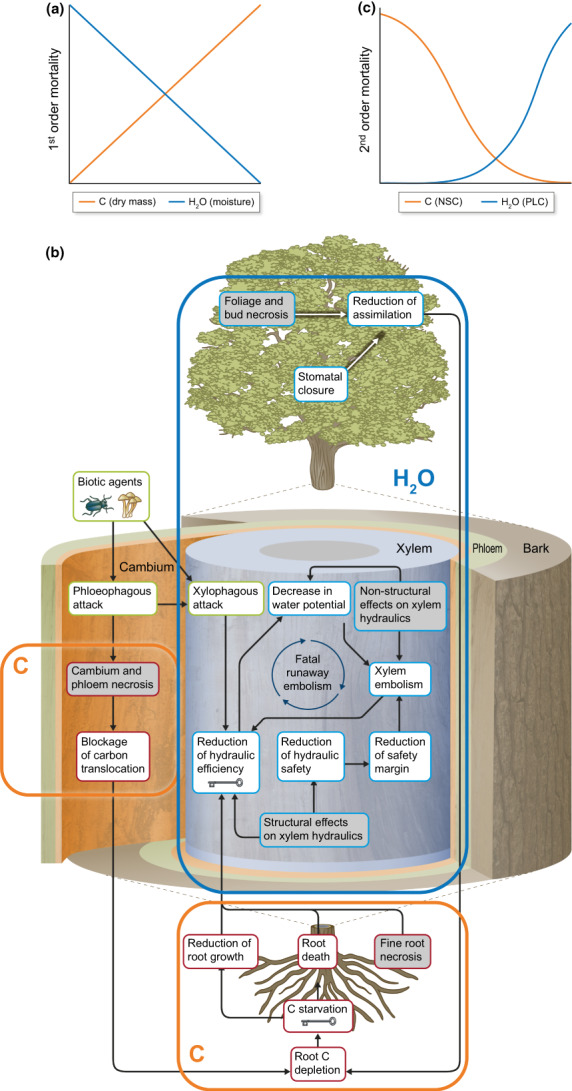
Plant carbon (C, orange) and water (H_2_O, blue) determine fire effects. (a) First‐order effects are the direct result of heat transferred to plant tissues (Michaletz & Johnson, 2007), as modulated by plant tissue properties (water and carbon content). Percent mortality increases with time integrated from ignition to cessation of fire (fire radiative energy density (FRED); Smith *et al*., 2017; Steady *et al*., 2019), which should approximate flame and smolder duration (Fig. 1e), which increase with leaf carbon (dry mass) and decrease with leaf water (moisture) content (Grootemaat *et al*., 2015; Bianchi *et al*., 2019). (b) Conceptual diagram illustrating the cascade of potential physiological responses to post‐fire injuries in plant roots, stems, and crowns adapted from Bär *et al*. (2019). Blue frame captures processes impacting the plant water cycle leading to reduction in hydraulic efficiency and ultimate hydraulic failure (fatal runaway embolism). Orange frames capture processes impacting the plant carbon cycle, leading to carbon starvation. (c) Second‐order effects are modulated by post‐fire water and carbon availability, along with integrity of the water and carbon uptake and transport systems (Hood *et al*., 2018; Bär *et al*., 2019). Mortality thresholds for hydraulic failure and carbon starvation are defined based on percent loss of conductivity (PLC, Hammond *et al*., 2019) and non‐structural carbohydrate (NSC) concentrations (Barker Plotkin *et al*., 2021), both of which are impacted by fire (Varner *et al*., 2009; Michaletz *et al*., 2012; West *et al*., 2016).

First‐order effects of fire on vegetation are the direct result of combustion and heat transfer to plant tissues (Michaletz & Johnson, 2007), and can be modulated by plant physiological characteristics. The same traits that influence fire behavior through controls on heat transfer also influence fire effects by modulating tissue temperatures and exposure times, particularly in low and mixed‐severity fire regimes where vegetation is not combusted and immediately killed (O'Brien *et al*., 2018; Varner *et al*., 2021). Percent mortality has been demonstrated to increase with fire radiative energy density (Smith *et al*., 2017; Steady *et al*., 2019), or time‐integrated radiative flux density from ignition to cessation of the fire. Since absorbed fire radiative energy is tied to flame and smolder duration (O'Brien *et al*., 2016), which increases with dry mass and decreases with moisture (Fig. 2e), we expect first‐order mortality to increase with dry mass and decrease with moisture content (Fig. 3a). This is consistent with significant increases in simulated canopy fuel consumption with reduced canopy fuel moisture, particularly under low‐wind scenarios (Sieg *et al*., 2017). While the physical relationship between moisture and flame and smolder duration should hold across species and ecosystems, further research is required to validate the relationship between moisture content and first‐order mortality, as tissues with higher moisture contents can also be more heat sensitive (Wright & Bailey, 1982).

When vegetation is not consumed, fire‐induced injuries, including hydraulic dysfunction, cambium and apical meristem necrosis, leaf and fine root necrosis, are proposed to influence whole‐plant carbon and water budgets resulting in hydraulic failure and/or carbon starvation (Fig. 3b; Midgley *et al*., 2011; Michaletz *et al*., 2012; Hood *et al*., 2018; Michaletz, 2018; Silva *et al*., 2018; Bär *et al*., 2019; Berenguer *et al*., 2021). This framework suggests that second‐order effects on live vegetation are driven, in part, by postfire water and carbon availability, along with integrity of the water and carbon uptake and transport systems (Fig. 3b,c; Michaletz & Johnson, 2007; Hood *et al*., 2018; Michaletz, 2018; Bär *et al*., 2019). Postfire environmental conditions and species‐specific traits determine whether resulting functional and growth limitations will lead to vegetation recovery or mortality (Bär *et al*., 2019; Sayer *et al*., 2020; Hood, 2021; Ruswick *et al*., 2021). For instance, traits such as water stress resistance are observed to differ between disturbance‐dependent and obligate sprouters in Mediterranean‐type climate regions (Pratt *et al*., 2012), with implications for postfire community composition. Franco *et al*. (2014) note that in neotropical savannas, where fire is common and accelerated fire frequencies and prolonged droughts are expected under climate change, the interplay between temperature optima and CO_2_ fertilization effects on photosynthesis, photorespiration, and respiration will define how much carbon is available for postfire plant growth and resprouting, determining the acclimation potential of a given species, plant community or ecosystem. Indeed, from Mediterranean to Tropical ecosystems, the balance between forest expansion and retreat, between seedling recruitment and mortality, has been attributed to species differences in drought sensitivity (e.g. embolism resistance, rooting depth, and stomatal regulation) and carbon balance (e.g. stomatal regulation, osmotic adjustment, and carbon allocation) (Keeley, 1998; Franco *et al*., 2014).

Drought and herbivory research, where tree mortality has a long history of study, has defined mortality thresholds for hydraulic failure in terms of percent loss of hydraulic conductivity (PLC; Hammond *et al*., 2019), and for carbon starvation in terms of nonstructural carbohydrate (NSC) depletion (Barker Plotkin *et al*., 2021). Evidence of increased PLC from laboratory experiments (Michaletz *et al*., 2012; West *et al*., 2016; Partelli‐Feltrin *et al*., 2021), as well as from forest fires (Bär *et al*., 2018), indicates that fire increases vulnerability to cavitation (Midgley *et al*., 2011; Hood, 2021). In addition, fire can decrease root NSC (Varner *et al*., 2009), reducing stores available to support metabolism, growth, and subsequent stress response, resulting in mortality independent of canopy damage. Together, this research suggests similar relationships for second‐order fire mortality (Fig. 3c) as for mortality from drought and herbivory, where sufficiently high PLC or low NSC results in mortality. This is consistent with recent work linking mortality and recovery from low‐intensity fire to prefire water status (van Mantgem *et al*., 2018; Partelli‐Feltrin *et al*., 2020) and concentrations of NSC (Zhu *et al*., 2012; Sayer *et al*., 2020). In addition, smoldering consumption can impact water uptake directly through root loss (O'Brien *et al*., 2010). In conifers, trade‐offs have also been documented between resistance to cavitation and to fire, likely mediated by allocation of carbon to either building thick bark or dense xylem, but not both (Resco de Dios *et al*., 2018). These trade‐offs have implications for woody vegetation recovery from fire under climate change, where drought conditions can predispose woody vegetation to hydraulic (Partelli‐Feltrin *et al*., 2020) and carbon (Sayer *et al*., 2020) limitations, impacting capacity to recover from fire‐induced injury and/or resprout. Indeed, intense postfire drought can also cause significant resprout mortality resulting from simultaneous loss of hydraulic conductivity and depletion of root starch (Pratt *et al*., 2014).

### 3. Importance of plant water and carbon dynamics

**Fig. 4:**
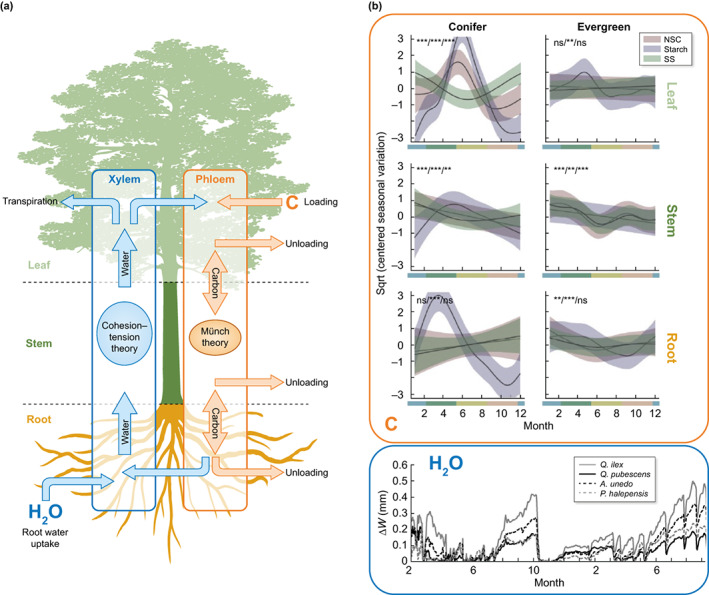
Plant carbon (C, orange) and water (H_2_O, blue) are constantly varying across organs (a), species and time (b). (a) Plant water (H_2_O, blue) and carbon (C, orange) exchange across organs (leaf, root, stem) by cohesion‐tension and Münch flow through xylem and phloem adapted from Zhou *et al*. (2020). (b, Carbon) Global patterns of seasonal variation (centered smooths, square‐root transformed mg g^−1^ dry mass) in total non‐structural carbohydrates (NSC), starch, and soluble sugars (SS) as a function of month, functional type (columns; conifer, evergreen angiosperm), and organ (rows; leaf, stem, root) adapted from Martínez‐Vilalta *et al*. (2016). (b, H_2_O) Seasonal course of tree water deficit (∆*W*, de‐trended stem diameter variation) for four species representing three different Mediterranean forest functional types: evergreen sclerophyllous (*Quercus ilex* L., *Arbutus unedo* L.); winter deciduous (*Quercus pubescens* Willd.); early successional, drought‐adapted conifer (*Pinus halepensis* Mill.) (adapted from Sánchez‐Costa *et al*., 2015).

Given the demonstrated role of plant water and carbon status in both fire behavior (Fig. 2) and effects (Fig. 3), accurately capturing their dynamics (seasonal variation, differences across species or functional types, with topography, and natural disturbance) will lead to improvements in fire behavior and effects models. Carbon and water status of live fuels change dynamically through time with environmental conditions and seasonal phenology (Baffoin *et al*., 2021). Variation in plant dry matter is driven by changes in organic molecules, largely NSC and lipids synthesized in the leaf and allocated to different plant organs (i.e. roots, stems, leaves, and reproductive structures) as needed for respiration and storage (Chapin *et al*., 1990). Diurnal variation results from daily synthesis and export patterns, while seasonal variation is largely driven by re‐allocation to meet seasonal demands associated with phenology or source limitation (e.g. drought‐induced reduction in photosynthesis; Martínez‐Vilalta *et al*., 2016). Changes in water content are driven by the interplay between soil water availability, physiological and morphological regulation, and atmospheric vapor pressure deficit through the soil–plant‐atmosphere continuum (Nelson, 2001; Macias Fauria *et al*., 2011). Water and carbon status differ across organs and over time as water moves from root to leaf *via* transpiration and as carbon is assimilated and transported across organs *via* the phloem (Fig. 4a, Zhou *et al*., 2020), and these dynamics differ by species and/or plant functional type. For instance, while evergreen conifers show strong seasonal variation in leaf NSC and dampened variation in stem NSC and water deficit (∆*W*), the opposite is true for evergreen angiosperms (Fig. 4b, Sánchez‐Costa *et al*., 2015; Martínez‐Vilalta *et al*., 2016). Topographic variation (microclimates, water availability, and soil resources available for growth) can also modify physiology, and fuel availability and loading, and has been shown to govern fire behavior and effects (Knapp *et al*., 1993; Krawchuk *et al*., 2016; Swann *et al*., 2022). Different functional strategies in relation to seasonal water deficit and disturbance, such as those defined by leaf lifespan (deciduous vs evergreen) and minimum water potential (Ackerly, 2004), will determine how these water and carbon dynamics manifest across species. Even within an individual species, plants can modulate the effects of variability in water resources by modifying leaf area, rooting depth, and/or stomatal conductance (Rambal, 1993). Among co‐occurring chaparral shrubs, differences in rooting depth can drive differential responses of leaf water potential to seasonal drought (Davis & Mooney, 1986; Smith & Richardson, 1990), and those with similar responses may use different modes of seasonal osmotic adjustment to accomplish changes in osmotic potential (i.e. shift in water volume vs solute concentration; Bowman & Roberts, 1985), with strong implications for LFMC. Species differences in drought response and associated leaf senescence and shedding have implications for litter accumulation and fuel‐bed flammability as well due to species variation in litter flammability and nonadditive effects in multi‐species litter mixtures (de Magalhães & Schwilk, 2012, 2021).

Climate and phenology drive seasonal change in these fire‐relevant traits, regulating water and NSC balances. Under future climate conditions, drought and phenological shifts can affect greenup, senescence, and susceptibility to mortality, all of which impact fuel load and flammability (Jolly *et al*., 2016). For instance, prefire drought increases the likelihood of mortality (van Mantgem *et al*., 2018; Partelli‐Feltrin *et al*., 2020) and of resprouting failure (Karavani *et al*., 2018; Resco de Dios *et al*., 2020). Topographic variation can be an important control on drought induced mortality and resulting fuel loads. For example, increased water availability in concave areas can mediate the increase in hydraulic stress typical at lower elevations (Tai *et al*., 2017). Prefire drought and heat stress may also diminish flowering and seed production, negatively impacting species with fire‐cued recruitment (Nolan *et al*., 2021). Heat susceptibility of tissues (Bär *et al*., 2021) and probability of vegetation survival can also be influenced by plant phenological stage during a burn (Trabaud, 1991; Knapp *et al*., 2009; Ruckman *et al*., 2012; Pratt *et al*., 2014), highlighting the importance of capturing seasonal variation in prediction of mortality (Bond & van Wilgen, 1996). Beyond drought, other natural disturbances can cause physical damage to vegetation (e.g. pathogens and herbivory), change in fuel loads and availability due to rapid mortality (e.g. bark beetles and tropical cyclones), or increased resource availability for plant growth (e.g. windfall), all of which can be incorporated into plant physiology models to understand their feedbacks on fire behavior and effects (Karp *et al*., 2021; Rouet‐Leduc *et al*., 2021; Fettig *et al*., 2022; Ibanez *et al*., 2022; Lee *et al*., 2022). Acknowledging dynamic physiology recognizes that fire behavior and its effects will vary, within and across species, with plant water status (Nolan *et al*., 2018), carbon dynamics (photosynthesis, respiration, allocation), phenology (Bär *et al*., 2021), species (McAllister & Weise, 2017; Nolan *et al*., 2018; Resco de Dios, 2020), and time (Fig. 4).

## IV. Integrating fine‐scale physiology with fire models

The wildland fire research community has called for the use of process‐based models to explore the potential mechanisms and interactions driving fire dynamics and effects and to conduct virtual experiments that allow for consideration of no‐analog future climate conditions (Michaletz *et al*., 2013; Hoffman *et al*., 2018; O'Brien *et al*., 2018). The demonstrated importance of plant water and carbon to fire behavior and effects, and their variability across species and time, underscore the value in applying plant carbon and water process models to explore the biophysical mechanisms linking vegetation to fire behavior and effects under present and future conditions.

Recent advances in whole‐plant modeling offer a promising framework for linking simulations of plant water‐ and carbon‐related processes to finer‐scale models of fire behavior and effects. Nolan *et al*. (2018, 2020) suggested a physiological basis to model LFMC for fire behavior applications using leaf water potential as a proxy. This approach allows for species‐specific variation with environmental drivers, while acknowledging the importance of capturing variation in leaf mass to account for decoupling of seasonal changes in the water and carbon contents that comprise LFMC. Mechanistic functional structural plant models (FSPMs) (Vos *et al*., 2010; Louarn & Song, 2020; de Vries, 2021), of 3D plant structure, environment, and physiological response, from gene to community scales, offer a potential solution to capture both water and carbon cycle processes. Functional structural plant models, which can simulate water and carbon flows across 3D plant compartments (root, stem, and leaf) in response to environmental conditions, have been demonstrated for use in simulation of both small plants and trees and include open‐source platforms that enable coupling with external modeling tools (e.g. Zhou *et al*., 2020).

**Fig. 5:**
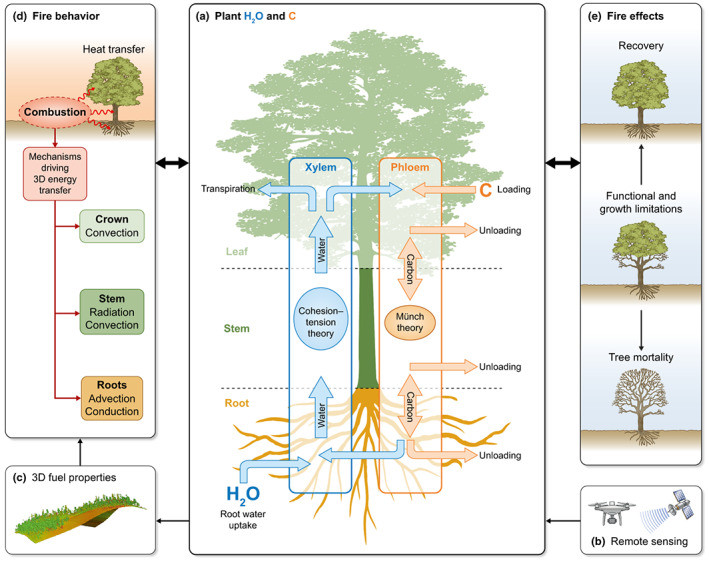
Plant carbon (C) and water (H_2_O) dynamics provide the mechanistic linkage between fire behavior and effects. In our conceptual framework, plant carbon and water process models (a; adapted from Zhou *et al*., 2020), which can be parameterized by remotely sensed estimates of canopy carbon and water contents (b; e.g. Dahlin *et al*., 2013; Singh *et al*., 2015), provide input to 3D fuel models (c; e.g. Pimont *et al*., 2016; Parsons *et al*., 2018). Detailed fuel properties are then exported to physics‐based fire models (d; adapted from O'Brien *et al*., 2018; Bär *et al*., 2019) that provide heat transfer estimates for input to the plant water and carbon model (a), which feeds into simulation of fire effects (e; adapted from Bär *et al*., 2019). These effects, along with remotely sensed estimates (b), drive subsequent plant water and carbon (a), feeding 3D fuels (c), and the cycle repeats.

Such a model could be coupled to fine‐scale fire behavior models using fuel modeling frameworks such as Fuel3D (Parsons *et al*., 2011), FuelManager (Pimont *et al*., 2016), STANDFIRE (Parsons *et al*., 2018), or FastFuels (Parsons *et al*., 2020). These frameworks can generate the 3D structure of canopy, mid‐, and under‐story vegetation for input to physics‐based fire models, such as FIRETEC or WFDS, allowing examination of within‐stand 3D fuel–fire interactions. Currently, meteorological or remotely sensed data (see the Remote sensing of plant water and carbon to inform process modeling section) can be used to set the moisture properties in these fuel models. Moisture is most often assigned a fixed value by the user for the course of the simulation across minutes to hours, and only FuelManager allows for assignment of alive vs dead status. As an advance to enable mechanistic evaluation of fine‐scale interactions between physiological and heat transfer processes and their influence on fire behavior and effects, we propose incorporation of a physiological process model to determine fuel condition based on physiological descriptions (plant water and carbon‐related processes) of individual plants (e.g. Cochard *et al*., 2021; Ruffault *et al*., 2022a,b). This could be achieved through use of a FSPM with remotely sensed parameterization (Fig. 5a,b) to inform 3D fuel properties (Fig. 5c). These properties would provide the necessary inputs to parameterize fire behavior models (Fig. 5d), the outputs of which could inform predictions of subsequent plant water and carbon dynamics (Fig. 5a). Model representations of these postfire physiological processes could then inform second‐order fire effects (Fig. 5e) through a process‐based mortality model (e.g. Michaletz & Johnson, 2008; Butler & Dickinson, 2010) and subsequent 3D fuel properties (Fig. 5c). This approach will require parallel advances in mechanistic modeling of whole‐plant fire injury processes to determine whether interacting injuries result in ultimate recovery, mortality, or functional limitations (Hood *et al*., 2018; Bär *et al*., 2019), which will be essential to determining subsequent fuel properties.

The individual plant (or within‐plant) detail of this approach will enable further exploration of the plant traits most critical to the process of, and sensitivity to, heat transfer, allowing for determination of scale‐relevant parameters. A model sensitivity approach varying plant physiological input parameters could be used to define functional groupings, based on species traits (response of tissue‐level water and carbon to environmental drivers) that influence fire behavior and effects, which may allow for more generalized response functions to reduce computational resource requirements for use at larger scales.

By directly linking combustion, physiological process, and fire‐injury plant‐mortality models to hydrologic and plant succession models in a spatially explicit way, this framework could potentially be extended to simulate landscape‐scale ecohydrology, and its response to fire disturbance under present and future climate conditions. By calculating transient heterogenous fuel moistures and temperatures based on incident radiation, heat fluxes, and topographic variation, this model extension could capture species‐ (or functional type‐) dependent responses to soil moisture at different depths. Landscape‐scale models of ecohydrology could use available downscaled climate reconstructions and models and spatially explicit maps of soil characteristics, topography, and aspect (e.g. Thornton *et al*., 2014; Soil Survey Staff, 2022) to simulate plant and fire response to landscape heterogeneity. This would allow plant water and carbon to vary spatially, influencing both fire behavior and vegetation mortality, which feedback to alter hydrology and plant succession, influencing ecosystem trajectory and response to subsequent fire disturbance. Such a framework could also allow for exploration of the role of other natural disturbance, alongside species composition and competitive interactions, in determining fuel load and the likelihood of future fire.

Another way to link fine‐scale mechanistic models to coarser scale outcomes is through metamodels and nested model design. These techniques aim to find the crucial aspects of the mechanism that would be propagated at a higher level of spatial or temporal coarseness. Traditional or machine‐learning metamodels can emulate nonlinear processes at scales where simulation would be unfeasible (Sparks *et al*., 2011; Huang *et al*., 2016). These can be integrated into existing coarse‐scale models to propagate fine‐scale understanding in a more computationally efficient manner (Lu & Ricciuto, 2019). A nested model design can allow for experimentation at relevant scales of physiology and mechanism to constrain coarser models or metamodels. For example, species trait‐based functional types of fire behavior and effects can be created and used at coarser scales; while separately simulating component species at scales relevant to physiology, combustion, or fire injury (Neilson *et al*., 2005). Bayesian methods can further allow for integrating multiple nested models with observational data to constrain the performance of coarse‐scale models along with the propagation of uncertainty (Talluto *et al*., 2016).

## V. Remote sensing of plant water and carbon to inform process modeling

Remote sensing of vegetation exploits the biophysical links between spectral information contained in the electromagnetic radiation reflected or emitted from the earth's surface and vegetation state, function, and dynamics. Optical data have been used to map LFMC using multispectral sensors at local (Marino *et al*., 2020), continental (Yebra *et al*., 2018a), and global (Quan *et al*., 2021) scales, producing mapping products at spatial resolutions from tens to thousands of meters. Visible spectrum reflectance is collected by many remote sensing platforms for vegetation monitoring purposes; but alone is insufficient for the prediction of LFMC. Addition of spectral data from the shortwave infrared (SWIR) region of the electromagnetic spectrum, currently collected by only a limited number of platforms, increases the accuracy of optical LFMC estimations (Yebra *et al*., 2018a). One challenge with direct optical estimation of fuel moisture is that satellite and airborne platforms output reflectance data as two‐dimensional coverages. In forested areas, these 2D data typically capture canopy conditions while obscuring understory characteristics (Yebra *et al*., 2018b), which are likely to differ widely from the overstory due to compositional and physiological differences. Similarly, these data are confounded by the presence of clouds, which are transient, yet exceedingly common, in many fire‐frequent areas of the world.

Acknowledgment of these challenges has led to research on the application of microwave remote sensing for direct and indirect fuel moisture content estimation (Fan *et al*., 2018; Wang *et al*., 2019). Typically, LFMC is estimated using radar‐derived vegetation optical depth, a proxy for vegetation water content (Moesinger *et al*., 2020). Radar systems operate in the microwave portion of the electromagnetic spectrum and can penetrate cloud cover and the upper canopy to detect the volume of liquid water in vegetation, offering an advantage relative to optical data. Nonetheless, a problem shared by direct microwave and passive optical measurements of LFMC in forests is the discrimination of signals related to the forest canopy, understory, surface litter, and soil (Gale *et al*., 2021). Merging microwave and optical data is a promising way forward. Rao *et al*. (2020) demonstrated that radar backscatter observations (from Sentinel‐1) directly enhanced LFMC predictability in comparison with only using optical reflectance (from Landsat‐8) for foliar, herbaceous and fine woody fuels. However, LFMC of denser woody fuels can only be estimated using longer wavelengths (i.e. L‐band radar; Tanase *et al*., 2015).

Despite these advances in mapping LFMC, little has been done to provide independent measures of dry mass and water content in the estimation of LFMC. To this end LMA (g m^−2^; also called dry matter content for remote sensing applications) and equivalent water thickness (EWT, g m^−2^) are appropriate target traits for optical sensing, capturing the effects of changing carbon allocation and moisture, respectively. Detection of EWT using optical sensors has a strong physical basis because water absorbs near infrared and short‐wave infrared radiation (Yebra *et al*., 2013). At the leaf scale, variation in LMA has a moderate influence on reflectance in the short‐wave infrared wavelengths (Feret *et al*., 2008) given the absorption features of structural properties found in leaves (Curran, 1989). For example, estimation of morphological and structural biochemical properties, including LMA, using reflectance spectroscopy of dried leaf material has been shown to be highly effective (e.g. Serbin *et al*., 2014). However, the presence of water in hydrated leaves can make the spectral estimation of dry matter content more challenging because water absorption masks the contribution of dry matter to the spectral response (Bowyer & Danson, 2004; Riano *et al*., 2005). Despite these challenges, the estimation of LMA at leaf and canopy scales has been shown to have reasonable accuracy using hyperspectral instruments (Ely *et al*., 2019; Chlus *et al*., 2020; Kamoske *et al*., 2021), likely due to covariance between EWT and LMA, as well as between leaf‐level functional properties and plant and canopy growth form and structure (Ollinger, 2011). Radiative transfer models have also been inverted using multispectral optical data to simultaneously estimate LMA and EWT and, therefore, LFMC (Yebra *et al*., 2013).

At smaller scales, techniques exist that allow for high‐temporal resolution vegetation carbon and water inventories. For instance, novel spectroscopic approaches leveraging high‐resolution spectra‐trait modeling (Serbin & Townsend, 2020; Burnett *et al*., 2021) provide the opportunity to more directly link spectral signatures with underlying physiology and plant condition (e.g. water and carbon content) in 2D (e.g. Dahlin *et al*., 2013; Singh *et al*., 2015) and 3D (Chlus *et al*., 2020; Kamoske *et al*., 2021). Likewise, dual‐wavelength lidar shows promise in moisture content estimation due to its ability to distinguish forest layers through ranging (Gale *et al*., 2021). These approaches are currently not available on space‐based platforms, limiting spatial coverage. However, these systems can be used together with ground‐based systems (e.g. imaging spectroscopy) to calibrate airborne and spaceborne hyperspectral (e.g. EnMAP, Guanter *et al*., 2016; SBG, Cawse‐Nicholson *et al*., 2021; OzFuel, ANU Institute for Space, 2021), thermal (ECOSTRESS, Anderson *et al*., 2021), and lidar (GEDI, Rishmawi *et al*., 2021) missions.

While current remote sensing LFMC products lack immediate predictive capability beneficial for fire management, they can be used to improve next‐generation process models directly (parameterization) or indirectly (benchmarking predictions). This will lead to improved simulations of fire behavior and spread under changing climate conditions. A challenge posed by the availability of remote sensing data is ensuring integration of data from disparate sources with existing databases for fuel characteristics and LFMC. New ‘big’ data systems are needed for standardized fusion of data and scalable dynamic updates to fuel data in a changing environment.

## VI. Conclusions and future directions

Fire behavior and effects are intrinsically associated with plant physiology through water and carbon cycles. Integration of plant water and carbon process models with fine‐scale fire models will allow for the representation of process‐level feedbacks between fire behavior and effects, enabling the use of virtual experiments to explore vegetation responses to global change scenarios (increased temperature and vapor pressure deficit, change in precipitation) and resulting fire outcomes. Recent advances in remote sensing, in combination with upcoming sensor deployments, will enable high‐resolution mapping of plant water and carbon status across regions and seasons, providing parameterization and benchmarking for next‐generation models. Several areas are ripe for development across the disciplines of plant physiology, fire modeling, and remote sensing to fully enable these advances in understanding and simulation of vegetation–fire interactions. Furthermore, while the research presented here focuses on woody plants (trees and shrubs), additional efforts are needed to understand the interactions between climate, fire, and physiology of other vegetation types that contribute to fire regimes (Knapp, 1985; D'Antonio & Vitousek, 1992; Taylor *et al*., 2014; Simpson *et al*., 2016). Parallel advances on all frontiers will be critical to meet the urgency of the wildfire crisis.

With respect to fire behavior, we need to better understand the impacts of plant carbon and water on heat transfer and their interaction with fuel structure beyond the leaf scale. For fire effects, more studies are necessary to disentangle the physiological impacts of fire on tissue‐level water and carbon status, along with interactions across organs to enable scaling to whole plant function and mortality (Hood *et al*., 2018; Michaletz, 2018; Kleynhans *et al*., 2021). We need benchmark datasets of physiological mortality mechanisms across organs, species, size class, life history, geographic region, season, and under climate change scenarios (Hood *et al*., 2018). New terrestrial lidar approaches for evaluating fuels and fire effects that are based on structure and visual‐spectrum imagery may be a useful approach for determining these benchmarks and understanding vertical heterogeneity in fire impacts on plant organs at these scales (Gallagher *et al*., 2021; Pokswinski *et al*., 2021).

Fire model implementation needs to address the nonlinear biophysical‐processes of whole‐plant heat transfer and resulting fire behavior and effects based on dynamic physiology, including refined fire‐effects predictions stemming from an improved understanding of mechanisms (Kleynhans *et al*., 2021). Advances in machine learning can provide estimations of unknown process and enhance computational efficiency but require us to understand underlying processes and diagnose cases of overfitting. Directly computing many of these dynamics may be increasingly tractable as computation progresses. We additionally need to understand how the interaction among these dynamics affects fire and tree mortality at scales relevant to human decision‐making. Using the inherent advantage of each scale of fire models, nested model design can work to incorporate new understanding at multiple scales (for example, plant physiology, stand management of plant density, landscape fire spread and suppression, and global carbon balance). Developing and validating nested model designs will be crucial to understand how each scale of organization influences the larger scale of organization.

Advancements in model implementation require finer spatial and temporal resolution observations of important processes to fire behavior and effects. Concerning remotely sensed drivers, we need to disentangle the numerous contributions to foliar reflectance signatures, along with advanced techniques for detection of subcanopy vegetation (Gale *et al*., 2021). Improved spatial and spectral resolution will reduce uncertainty in characterization of water and carbon status across regions and seasons, and better statistical methods will make outcomes more robust across larger areas and disparate biomes (Yebra *et al*., 2013). Advancements in data reduction and/or model ingestion will be needed to allow effective use of this higher resolution data. Ground‐based experiments and observations can be used to better describe vegetation–fire interactions, and every prescribed burn provides an opportunity for data collection and model validation.

Nelson (2001) suggested that ‘the complexity of the task may have precluded all attempts to develop a reasonably complete physics‐ and physiology‐based model’ of live fuel moisture. With an increasing wealth of drought physiology research, the advent of next‐generation models, and new spaceborne sensor deployments on the horizon, we are finally poised to tackle the problem of providing a dynamic and mechanistic description of fire behavior and effects through vegetation processes. Doing so will better equip us and the ecosystems we depend on to survive and thrive in a future made uncertain by global change.

## Competing interests

None declared.

## Author contributions

This manuscript was the outcome of a three‐day workshop hosted by Los Alamos National Laboratory in January 2021. LTD, AKJ and RRL planned and designed the workshop. ADC facilitated the workshop. LTD, AKJ, ALA, MRG, JKH, CMH, SMH, ELL, WMJ, RHN, JJO, RAP, FP, JR, SPS, SS, JKS, NSS, DRW, CX and NY gave presentations at the workshop. J‐LD, VRD, and MY contributed to presentations but were unable to attend. LTD, AKJ, and ADC moderated Q&A sessions. LTD, AKJ, RRL, ALA, JKH, WMJ, ELL, JJO and SPS moderated breakout sessions. LTD, AKJ, RRL, IA, ALA, ADC, MRG, JKH, CMH, SMH, MDH, WMJ, AJ, ELL, WM, STM, RHN, JJO, RAP, RP‐F, FP, JR, KAS, ES‐F, SPS, SS, JKS, CHS, NSS, DRW, MW, CX and NY participated in the workshop. LTD and AKJ led manuscript development and writing. AB and ZJR were consulted for subject matter expertise on the manuscript. All authors contributed to manuscript writing and editing.
